# Optimization of Anti-SARS-CoV-2 Treatments Based on Curcumin, Used Alone or Employed as a Photosensitizer

**DOI:** 10.3390/v14102132

**Published:** 2022-09-27

**Authors:** Luisa Zupin, Francesco Fontana, Libera Clemente, Violetta Borelli, Giuseppe Ricci, Maurizio Ruscio, Sergio Crovella

**Affiliations:** 1 Institute for Maternal and Child Health IRCCS Burlo Garofolo, 34137 Trieste, Italy; 2 Division of Laboratory Medicine, University Hospital Giuliano Isontina (ASUGI), 34129 Trieste, Italy; 3 Department of Life Science, University of Trieste, 34127 Trieste, Italy; 4 Department of Medicine, Surgery and Health Sciences, University of Trieste, 34129 Trieste, Italy; 5 Biological Science Program, Department of Biological and Environmental Sciences, College of Arts and Sciences, University of Qatar, Doha 2713, Qatar

**Keywords:** photodynamic therapy, curcumin, SARS-CoV-2, Vero E6 cells, in vitro infection

## Abstract

Curcumin, the bioactive compound of the spice *Curcuma longa*, has already been reported as a potential COVID-19 adjuvant treatment due to its immunomodulatory and anti-inflammatory properties. In this study, SARS-CoV-2 was challenged with curcumin; moreover, curcumin was also coupled with laser light at 445 nm in a photodynamic therapy approach. Curcumin at a concentration of 10 μM, delivered to the virus prior to inoculation on cell culture, inhibited SARS-CoV-2 replication (reduction >99%) in Vero E6 cells, possibly due to disruption of the virion structure, as observed using the RNase protection assay. However, curcumin was not effective as a prophylactic treatment on already-infected Vero E6 cells. Notably, when curcumin was employed as a photosensitizer and blue laser light at 445 nm was delivered to a mix of curcumin/virus prior to the inoculation on the cells, virus inactivation was observed (>99%) using doses of curcumin that were not antiviral by themselves. Photodynamic therapy employing crude curcumin can be suggested as an antiviral option against SARS-CoV-2 infection.

## 1. Introduction

Turmeric, from *Curcuma longa*, is a spice that is widely used in southern Asia, especially in India. Due to its beneficial therapeutic properties, it has been employed for years in traditional medicine [1] for wound healing, the treatment of respiratory conditions and liver and skin disorders, as well as for antimicrobial purposes. Moreover, many of its properties, such as its anti-inflammatory, anti-diabetic, anti-cancer, and anti-aging activities, have been described [1]. Different bioactive compounds, including curcumin (diferuloylmethane, a natural polyphenol), desmethoxycurcumin, and bisdemethoxycurcumin are present in this spice [2].

Currently, curcumin is part of the nutraceutical market, which has been attracting great attention and popularity due to the increasing interest of the general public in natural products [3].

Several studies have reported the beneficial effects of curcumin as an adjuvant in the management of pediatric conditions such as inflammatory bowel disease [4], neuroblastoma [5], acute-onset neuropsychiatric syndrome [6], oral mucositis [7], asthma [8], and juvenile idiopathic arthritis-associated uveitis [9]. Curcumin showed also in vitro antitumoral activity against pediatric tumor cells, such as epithelial cancer liver cells [10], and rhabdomyosarcoma cells [11], when employed as a photosensitizer in a photodynamic therapy experimental setting.

Moreover, curcumin is widely known for its antimicrobial properties [12,13]. Curcumin possesses broad-spectrum antiviral actions, being active against RNA viruses, such as human immunodeficiency virus 1, zika virus, dengue virus, chikungunya virus, vesicular stomatitis virus, enterovirus 71, human respiratory syncytial virus, norovirus, and DNA viruses including herpes simplex virus 2, Kaposi’s sarcoma-associated herpesvirus, and human adenovirus [14]. Intriguingly, despite the wide range of susceptible viruses, the mechanism of action seems to be strain-specific, with different effects depending upon the virus or on cellular functions, resulting in the hampering of viral attachment, entry, replication, and egress, as well as presenting a direct effect on viral proteins, as extensively reviewed by Jennings and Parks [14].

Recently, curcumin was used to fight severe acute respiratory syndrome coronavirus 2 (SARS-CoV-2), showing a virus-neutralizing effect on Vero E6 and Calu-3 cells after 1–2 h of incubation [15,16,17], also when curcumin was administered prior to or post-viral infection [15,16]. A possible mechanism of action could be related to the inhibition of binding between SARS-CoV-2 and its receptor angiotensin-converting enzyme 2 (ACE2), as suggested by Goc et al. [15]. Another mode of activity could be exerted through the inhibition of the RdRP viral complex, as observed by Goc et al. in another study [18].

Curcumin encapsulated in polysaccharide nanoparticles also inhibited the inflammatory response induced by the spike protein in vitro on Huh7.5 and A549 epithelial cells [19].

A photodynamic approach using curcumin nanoparticles as a photosensitizer of blue laser light was also previously tested [20]. In a study conducted by Pourhajibagher et al., human plasma containing SARS-CoV-2 was mixed with curcumin nanoparticles, irradiated, and then inoculated on Vero E6 cells. The authors showed an inhibitory effect on the virus, assessed in terms of cell survival after treatment, compared to cells exposed to the non-treated virus.

Finally, nanocurcumin formulations were also effective in clinically improving the symptomatology of COVID-19 [21,22,23,24,25,26,27,28,29,30,31], although in patients it was difficult to determine whether the effects were related to the anti-inflammatory properties of curcumin [1] or to direct antiviral action [15,16,17,18,19].

In this study, antiviral approaches employing curcumin alone and as a photosensitizer in combination with blue laser light were investigated and optimized on a delta strain of SARS-CoV-2 in an in vitro model of infection.

Different settings were employed using reduced doses of curcumin compared to those used in previous works, as well as reduced treatment times [15,16,17,18,19]: (1) the incubation of the virus with curcumin prior to its inoculation on cell culture; (2) the treatment of cells with curcumin prior to infection; (3) the treatment of SARS-CoV-2-infected cells.

Moreover, a photodynamic experimental approach was set up, in which the mix of virus and curcumin was irradiated with a laser light at 445 nm and then transferred to cell cultures. In this setting, only raw curcumin was employed, whereas the only previous PDT study conducted against SARS-CoV-2 exploited a curcumin nanoformulation [20].

## 2. Materials and Methods

### 2.1. Vero E6 Cells and SARS-CoV-2 Detection

Epithelial Vero E6 cells (ATCC CRL-1586, *Cercopithecus aethiops* normal kidney) were maintained in MEM supplemented with 10% fetal bovine serum (FBS), 2 mM glutamine, and 100 U/mL penicillin/streptomycin (Euroclone, Pero, Italy). During the infection, the medium was supplemented with 2% FBS. The cells were seeded on 24 multiwell plates at a density of 5 × 10^4^ cells/well, and they were utilized in the experiments a day later.

SARS-CoV-2, previously isolated at the San Polo Hospital (ASUGI, Monfalcone, GO, Italy), was tested for the delta variant with the REALQUALITY SARS-CoV-2 AIM Variants (detecting AY.1/AY.2, B.1.617.2/B.1.617.1/B.1.617.3, B.351/B.351.2/B.351.3 strains, AB Analitica, Padua, Italy) and was positive for B.1.617.2/B.1.617.1/B.1.617.3 strains, identifying it as a Delta variant.

The virus was diluted at a multiplicity of infection (MOI) of 0.1 and mixed with different concentrations of curcumin from *Curcuma longa* (Turmeric, C1386, Merck KGaA, Darmstadt, Germany): 10, 5, 1, 0.5, 0.1, 0.05 μM (setting 1). After 5 min, the mix was inoculated in the Vero E6 cells for 1 h at 37 °C, then the supernatants were discharged, the cells were washed in phosphate buffer saline (PBS), and new medium was added.

The cells were monitored for 7 days and the RNA viral load in the supernatants was determined on days 4 and 7. Briefly, 15 μL of supernatants were harvested, mixed with 45 μL of water, and submitted to thermolysis (98° for 3′ and 4° for 5′). The RNA viral load was determined via real time PCR, targeting the N gene (CDC primers and probe: forward primer TTA CAA ACA TTG GCC GCA AA, reverse primer: GCG CGA CAT TCC GAA GAA, probe: FAM-ACA ATT TGC CCC CAG CGC TTC AG-BHQ1, Eurofins, Luxembourg) with the Luna^®^ Universal Probe One-Step RT-qPCR Kit (New England Biolabs, Ipswich, MA, USA) on the 7500 Fast Real-Time PCR system (Thermo Fisher Scientific, Waltham, MA, USA, protocol; 50 °C for 10′, 95 °C for 1′, and then 40 cycles at 95 °C for 10″ and 60 °C for 30″). The quantification was carried out using a nCoV-CDC-Control Plasmid as a standard (Eurofins).

### 2.2. Evaluation of Curcumin’s Mechanisms of Action

After the determination of the minimum dose of curcumin that was able to completely inhibit SARS-CoV-2 replication (10 μM), the RNAse protection assay was performed. After curcumin treatment, 15 μL of the treated and non-treated SARS-CoV-2 solution were hydrolyzed with RNAse enzymes (1 μg of Ribonuclease A R4875, Merck KGaA) for 30 min at 37 °C. Then, the samples were submitted to thermolysis, and RNA was quantified as described above.

To determine whether curcumin was also effective as a prophylactic treatment or as a potential cure for infected cells, two different settings were employed:

- The cells were pretreated with curcumin for 1 h, then curcumin was removed, and the cells were inoculated with SARS-CoV-2 for 1 h (setting 2, curcumin prophylactic treatment of cells).
- The cells were inoculated with SARS-CoV-2 for 1 h, then the supernatants were removed, and the cells were treated with curcumin for 1 h (setting 3, curcumin treatment of SARS-CoV-2 infected cells).

At the end of the absorption period, the supernatants were discharged, the cells were washed in PBS, and new medium was added. The viral load was quantified in the supernatants as described above (Section 2.1).

### 2.3. Photodynamic Treatment

Photodynamic treatment (PDT), employing curcumin as a photosensitizer, was also performed. The virus was mixed with curcumin with doses that had not exhibited antiviral effects (5, 1, 0.5, 0.1, and 0.05 μM) and then irradiated with a class IV diode laser (K-Laser Blue series, K-laser d.o.o., Sežana, Slovenia) with an irradiation protocol, which had been previously determined to be non-cytotoxic and non-antiviral itself (445 nm λ, irradiance 0.25 W/cm^2^, fluence 15 J/cm^2^, in a continuous wave). At the end of the irradiation process, the virus-curcumin mix was transferred to cell culture for 1 h at 37 °C. Then, the supernatants were eliminated, the cells were washed in PBS, and new medium was added (setting 4, PDT treatment of SARS-CoV-2). The RNA viral load in the supernatants was determined 7 days post-PDT.

**Figure 1:**
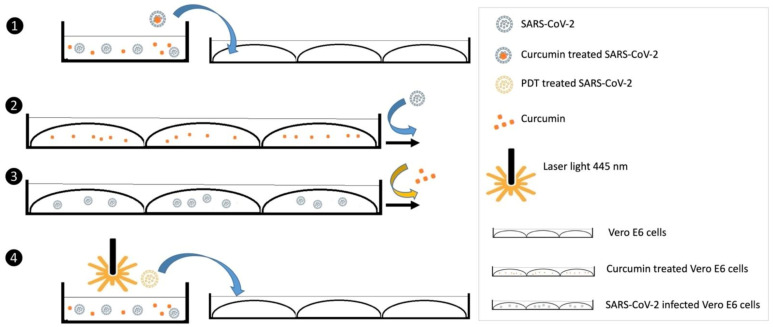
A graphical representation of the experimental settings employed: (1) the incubation of the virus with curcumin prior to its inoculation on cell culture (2) curcumin prophylactic treatment of cells (3) curcumin treatment of SARS-CoV-2 infected cells (4) curcumin based photodynamic treatment of SARS-CoV-2.

Figure 1 presents a schematic representation of the experimental settings employed in this study.

### 2.4. Viability Evaluation

To assess cell survival, on the 7th day post-infection/treatment, the viability of the cells was assessed with crystal violet staining. The supernatants were removed, and the cells were stained with crystal violet (10% in phosphate buffer saline—PBS) solution. After 30′, the cells were washed with distilled water and air-dried. Then, 200 μL of lysis solution (1% dodecyl sodium sulfate in PBS) were added to the wells for 30′, they were diluted in water, and the absorbance was read at 600 nm.

### 2.5. Statistical Analysis

The Mann–Whitney (MW) and Kruskal–Wallis (KW) test corrected for multiple comparisons with Dunn’s test were used to compare the different conditions tested on R statistical software. Each experimental setting was performed in at least 8 replicates on 2 independent days.

## 3. Results

When curcumin was mixed with the SARS-CoV-2 suspension and immediately inoculated to a monolayer of Vero E6 cells, it was able to completely block the viral replication (>99% of reduction). The inhibition was achieved with curcumin at 10 μM, whereas the other concentrations (i.e., 5, 1, 0.5, 0.1, and 0.05 μM) tested did not limit SARS-CoV-2 infectivity.

Our observations revealed a very rapid inactivation of the virus. Indeed, the virus and curcumin were mixed and inoculated after only 5 min into the cells without prior incubation, and a long-term antiviral effect was also observed, as highlighted by the lack of virus amplification on day 7 after treatment/inoculation. 

**Figure 2:**
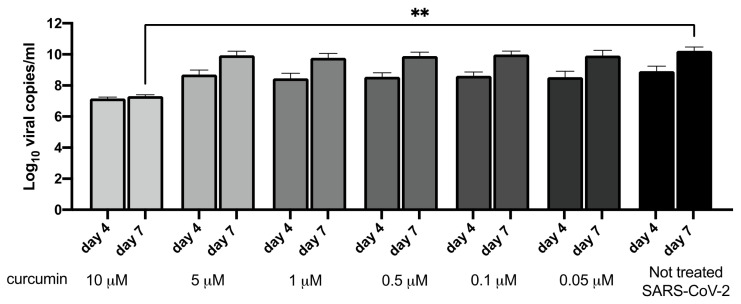
Effect of different concentrations of curcumin (10, 5, 1, 0.5, 0.1, and 0.05 μM) on SARS-CoV-2 viral load on days 4 and 7 post-treatment/infection. The viral load is expressed as Log_10_ viral copies/mL. Result from Kruskal–Wallis tests, adjusted for Dunn’s multiple comparisons test, are displayed. ** *p*-value < 0.01.

The RNA viral load quantified in the supernatants of the cells infected with curcumin-treated virus (10 μM) remained at 10^7^ RNA viral copies/mL on both day 4 and day 7, whereas in the Vero E6 cells incubated with the non-treated virus the viral load increased at 10^8^ RNA viral copies/mL on day 4 and on 10^10^ RNA viral copies/mL on day 7 (virus treated with 10 μM curcumin versus non-treated SARS-CoV-2—day 7, KW test adjusted *p*-value = 0.007; Figure 2).

With the aim of defining which possible step of viral replication was inhibited by curcumin, the substance at the efficacious concentration of 10 μM was tested in a prophylactic approach (setting 2), and on an already infected culture (setting 3), but no antiviral effect was observed (data not shown).

**Figure 3:**
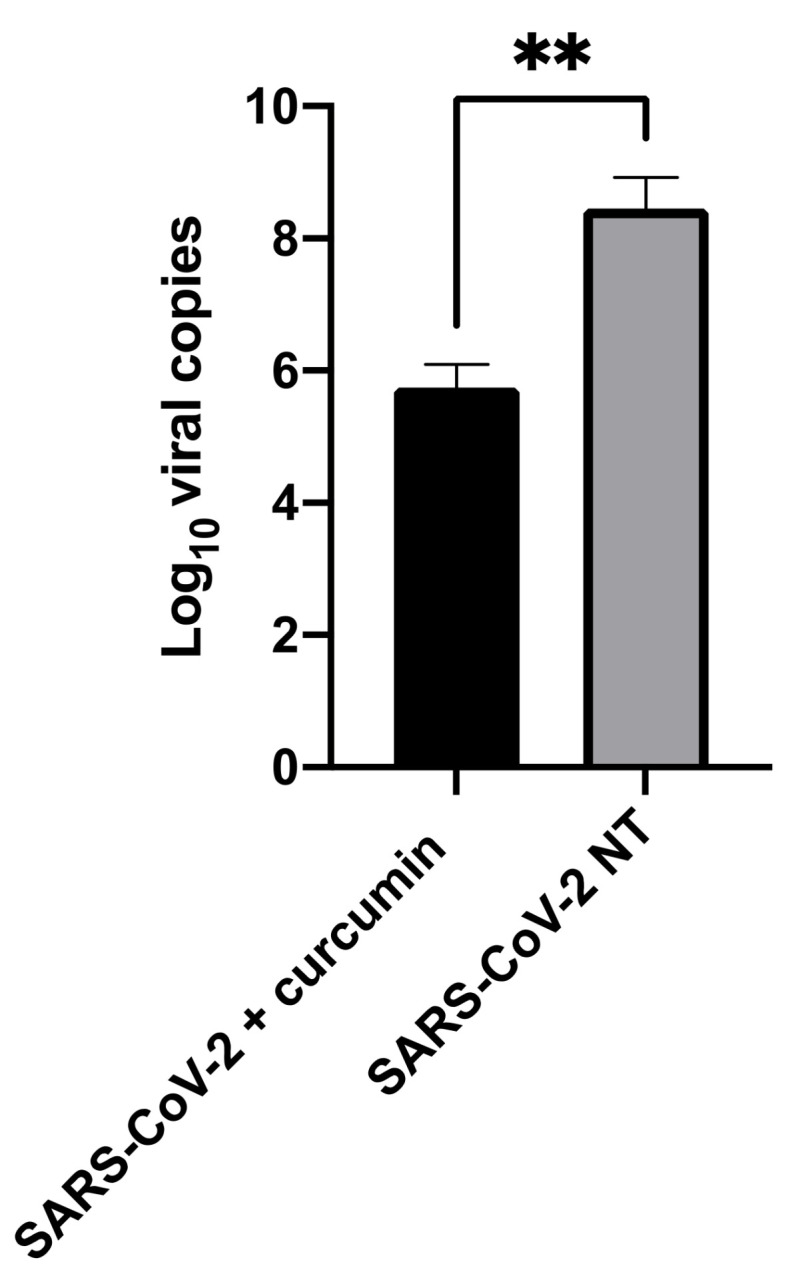
The effect of curcumin (10 μM) on virion integrity, tested by means of the RNAse protection assay. Non-treated (designed as SARS-CoV-2 NT) and treated viruses (designed as SARS-CoV-2 + curcumin) are displayed. The viral load is expressed as Log_10_ viral copies. Results from Mann–Whitney tests are shown. ** *p*-value < 0.01.

In an attempt to determine a possible mechanism of action, the RNAse protection assay was performed. In this assay, when the viral RNA is released into the solution from virions not presenting an intact envelope, it is degraded by the RNase, thus decreasing the viral load, whereas when the RNA is protected by the envelopes of intact virions, RNase cannot disrupt it and the viral load is not affected by the RNAse treatment [32]. In our experiments, a significant reduction in RNA was observed, from 2.85 × 10^8^ to 5.6 × 10^5^ RNA viral copies/mL (MW test *p*-value = 0.01, Figure 3) when the virus was treated with curcumin (10 μM), suggesting that curcumin may be able to partially disrupt virions.

In an attempt to decrease the effective virucidal concentration of curcumin, we tested the photodynamic strategy by delivering blue laser light onto the mixed suspensions of the virus and curcumin (at concentrations that did not present a direct viral effect by themselves, i.e., <10 μM), then transferring them onto a monolayer of Vero E6 cells for 7 days.

**Figure 4:**
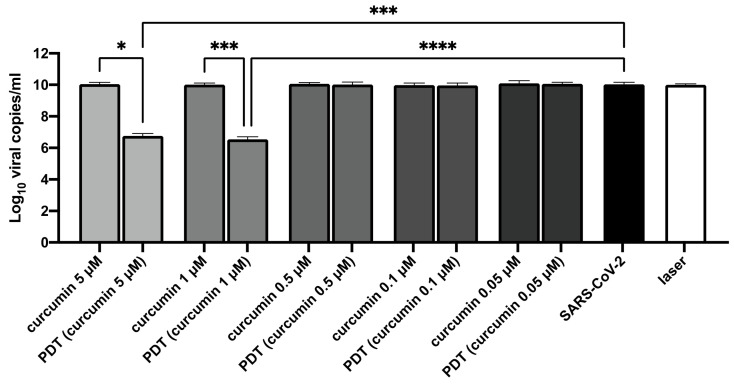
Results of photodynamic treatment (PDT) with different concentrations of curcumin (5, 1, 0.5, 0.1, and 0.05 μM) on SARS-CoV-2 infectivity (viral load) on day 7 post-treatment/infection. The data obtained through the irradiation of the virus without curcumin (indicated as “laser” in the figure), the treatment of the virus with curcumin without irradiation (designed as “curcumin”), and the non-treated virus (not irradiated and not challenged with curcumin, indicated in the figure as “SARS-CoV-2”) are also reported. The viral load is expressed as Log_10_ viral copies for mL. Results of Kruskal–Wallis test, adjusted for Dunn’s multiple comparisons test, are displayed. **** *p*-value < 0.0001; *** *p*-value < 0.001; * *p*-value < 0.05.

Interestingly, a >99% reduction in the viral load was observed in the supernatants from the cells inoculated with the PDT-treated virus (curcumin at 5 and 1 μM) compared to the wells in which Vero E6 cells were challenged with non-treated SARS-CoV-2 (SARS-CoV-2 treated with PDT—5μM curcumin versus non-treated SARS-CoV-2, KW test adjusted *p*-value = 0.0004; SARS-CoV-2 treated with PDT—1 μM versus non-treated SARS-CoV-2, KW test adjusted *p*-value = 0.000003; Figure 4), and compared to the cells infected with the virus challenged with only curcumin (SARS-CoV-2 treated with PDT—5 μM curcumin versus 5 μM curcumin treated SARS-CoV-2, KW test adjusted *p*-value = 0.03; SARS-CoV-2 treated with PDT—1 μM curcumin versus 1 μM curcumin-treated SARS-CoV-2, KW test adjusted *p*-value = 0.001; Figure 4).

Moreover, the viability at 7 days post-treatment/infection was assessed.

**Figure 5:**
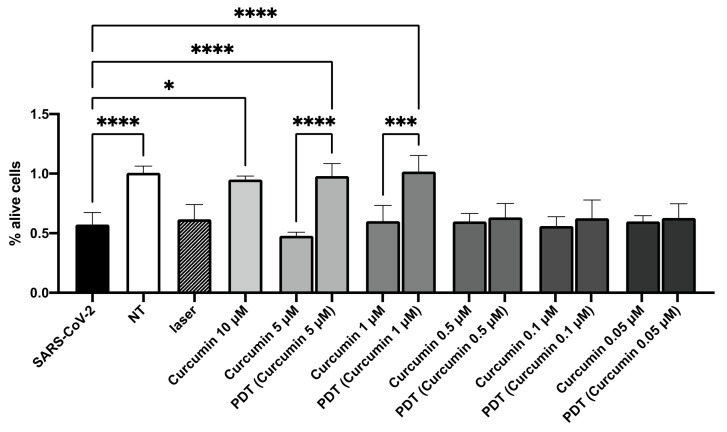
Viability at 7 days of the cells inoculated with curcumin-treated virus (indicated as 2curcumin”), PDT-treated virus (indicated as “PDT curcumin”), or untreated virus (indicated as “SARS-CoV-2”). Non-infected cells (indicated as NT) and cells infected with irradiated virus without curcumin (indicated as “laser”) are also shown. The data are presented as a percentage of living cells relative to the non-treated cells (NT). Results from Kruskal–Wallis test, adjusted for Dunn’s multiple comparisons test, are displayed. **** *p*-value < 0.0001; *** *p*-value < 0.001; * *p*-value < 0.05.

The survival of cells inoculated with non-treated SARS-CoV-2 dropped to 50% after 7 days. Nevertheless, the results obtained for the cells infected with the virus treated with curcumin alone at 10 μM, and the PDT-treated virus (with curcumin at 5 and 1 μM) were comparable to those obtained for the non-infected cells (SARS-CoV-2 versus virus treated with 10 μM curcumin, KW test adjusted *p*-value = 0.05; SARS-CoV-2 versus virus treated with PDT—5 μM curcumin, KW test adjusted *p*-value = 0.0000001; SARS-CoV-2 versus virus treated with PDT—1 μM curcumin, KW test adjusted *p*-value = 0.00000005; SARS-CoV-2 treated with 5 μM curcumin versus virus treated with PDT—5 μM curcumin, KW test adjusted *p*-value = 0.0000000001; SARS-CoV-2 treated with 1 μM curcumin versus virus treated with PDT—1 μM curcumin, KW test adjusted *p*-value = 0.0005; Figure 5).

## 4. Discussion

Despite the huge efforts of the vaccination campaigns against SARS-CoV-2, the virus is still spreading among the vaccinated and non-vaccinated populations, mostly due to the omicron variant [33]. Although the hospitalization rate of vaccinated individuals and the percentage of severe COVID-19 manifestations have decreased, the total number of SARS-CoV-2-positive individuals is still increasing [34].

Curcumin has been administered to COVID-19 patients with some evident benefits; indeed, nanocurcumin formulations are able to positively act on chills, cough, and smell and taste perception [21], as well as improving O_2_ saturation [24], while decreasing reactive protein C [21] and increasing lymphocyte counts [21,26].

Moreover, nanocurcumin was able to immunomodulate the inflammatory response, decreasing the levels of serum IL-17 [23], IFN-γ [22,23], IL-1 β, IL-6 [22,31], and TNF-α [22] and increasing those of IL-4 and TGF-β, as well as reducing the gene expression of *TBX21*, and *FOXP3* [23]. Nanocurcumin also promotes T-reg cells, increasing the expression level of FoxP3, IL-10, IL-35, and TGF-β (both mRNA and protein) in the same patients compared before and after treatment [30]. A significant decrement of T helper 17 (Th17) cells, with lower levels of Th17-cell-correlated factors and cytokines, was observed in COVID-19 patients treated with curcumin [29].

A combination of curcumin, quercetin, and vitamin D3 supplementation has been reported as adjuvating the recovery of COVID-19 patients. Indeed, at day 7, 60% of the treated patients obtained negative results at molecular tests and their symptoms had resolved, compared to 15% and 40% of patients in the placebo arm, respectively [25]. Curcumin [28] and a combination of curcumin/piperine [27] accelerated recovery from COVID-19 symptoms (fever, cough, sore throat, and breathlessness), along with better clinical outcomes and the ability to maintain a good oxygen saturation. Treatment with Sinacurcumin soft gel (containing curcuminoids formulated in nanomicelles) reduced the length of hospitalization (from 9 to 5 days), resulting in a complete recovery in 10 cases (a total of 21 patients) compared to 3 cases (a total of 20 patients) in the placebo group, in which a deterioration of symptoms was also observed in 8 patients [28].

These studies [21,22,23,24,25,26,27,28,29,30,31] showed that curcumin possesses an immunomodulatory effect on the host immune system, decreasing inflammation, and should be considered as an interesting adjuvant to the treatment of patients undergoing the COVID-19 cytokine storm and in severe COVID-19 cases, as well as in children. Indeed, multisystem inflammatory syndrome in children (called MIS-C) can occur, characterized by high fever, systemic inflammation, and multiorgan disfunction, which may also present with cardiac involvement [35].

Our in vitro study confirmed the findings in the literature which described the effectiveness of curcumin against SARS-CoV-2, although our data highlighted that the curcumin concentration and time of treatment can be reduced [15,16,17,18,19].

Indeed, a very short time of treatment was able to inhibit viral replication at a concentration of 10 μM, a quantity that is not harmful for cells, whereas when looking at the other lower curcumin concentrations tested, i.e., 5, 1, 0.5, 0.1, and 0.05 μM, no antiviral effect was observed. The same concentrations were also safe for human-derived cell lines, as we previously observed on HaCaT skin keratinocytes [12].

Our results are in agreement with the findings of Bormann et al. [17], showing a reduction of cytopathic effects in Vero E6 and Calu-3 cells 48 h after treatment/inoculation, leading to a complete inhibition at 15.6 μg/mL (42 μM) and an efficacious concentration (EC)_50_ of 7.9 μg/mL (21 μM). These findings were confirmed by assessing the RNA levels in supernatants, determining a 100% inhibitor dose at 125 μg/mL (339 μM) with an EC_50_ of 14 μg/mL (38 μM). The efficacious curcumin concentrations used by Bormann et al. were higher than those employed in our study (10 μM), as was the incubation time, at 1 h versus a few minutes, whereas the number of virions treated was on the same order of magnitude as that in our study. Our data are also in accordance with a study by Marín-Palma et al. [16], who tested a 1 h co-treatment approach, showing an antiviral effect—although the efficacious concentration was higher—with an EC_50_ of 3.57 μg/mL (9.69 μM) at 48 h post-infection/treatment and a 92% reduction in viral titer with curcumin at 10 μg/mL (27.14 μM).

With the aim of determining whether curcumin activity may affect virion integrity, the RNase protection assay was performed. When curcumin was added to the virus suspension, a 3 Log reduction of intact virions compared to non-treated SARS-CoV-2 was observed. These data suggest that the antiviral action of curcumin on SARS-CoV-2 may occur in part due to an impairment of envelope integrity, leading to the disruption of the viral particles and the release of RNA. The subsequent RNase treatment eliminated the released viral RNA, resulting in a low quantity of RNA in the curcumin-treated samples. 

Another mechanism of action of curcumin could be related to the inhibition of SARS-CoV-2 binding to host receptors. Indeed, an elegant study by Goc et al. [15] showed that curcumin was able to inhibit the binding between the SARS-CoV-2 spike protein and the ACE2 receptor, with a maximal effect observed at 100 μg/mL (271 μM). Moreover, curcumin decreased the binding and the entry of pseudovirus presenting the spike SARS-CoV-2 protein on hACE2/A549 cells when the curcumin was preincubated with the pseudovirus for 1 h, without prior incubation, and when curcumin was added after 1 h of infection. The doses tested ranged from 2.5 to 100 μg/mL (7–271 μM), with a greater effect observed at the highest doses. The molecular effect leading to this observation was not elucidated by the authors [15]; however, it has been previously shown that curcumin, as a lipophilic molecule, can induce the morphological modification of membranes, altering their fluidity [36] and thus affecting the fusion between hepatitis C virus and target cells [37].

Another mode of activity could rely on the inhibition of the RdRP viral complex (composed of non-structural proteins—nsp12/nsp7/nsp8) of both SARS-CoV-2 and the Omicron variant (curcumin at 0.1 mg/mL), as observed by Goc et al. in another study [18].

In an attempt to understand whether curcumin could also be useful in different experimental settings, it was tested on already-infected cells (1 h) or prior to infection; however, no antiviral effect was observed. In contrast with these findings, Marín-Palma et al. [16] reported the antiviral activity of curcumin when administered prior to or post-infection; however, the treatment durations were quite prolonged, 24 h before viral infection and 48 h after infection, compared to 1 h of treatment, which yielded negative results in our study; moreover, they employed higher concentration of curcumin (27 μM) compared to the efficacious one used in the current study.

Our negative results may suggest that, when employing short time of curcumin incubation (1 h) post- or prior to infection, curcumin may not be able to efficaciously interact with replication machinery when the virus is inside the cells, or the molecule may not accumulate within the cells, rendering them more resilient to infection. On the other hand, a long time of treatment (above 1 h) presents limited translatability to a real in vivo setting.

This lack of effects could be due to the hydrophobic characteristics of curcumin [38] and to its limited ability to enter cells in the formulation used in our experimental setting. Indeed, this hypothesis was confirmed by a study by Moustapha et al. [39] showing a 1:20 ratio between the curcumin inside the cells and in the extracellular medium. Regrettably, the therapeutic potential of curcuminoids has been limited so far by their low solubility in aqueous solutions, their poor bioavailability, and their suboptimal pharmacokinetic features [1]. With that being said, different strategies employing the use of curcumin nanoformulations have been prompted in order to overcome solubility and bioavailability issues, by exploiting curcumin loaded in micelles, liposomes, solid lipid nanoparticles, nanoemulsions, quantum dots, cyclodextrins, chitosan, graphene nanocomposites, mesoporous particles, and polymeric and metallic nanoparticles, to cite only some possibilities [40].

Indeed, Sharma et al. employed curcumin encapsulated in polysaccharide nanoparticles, showing that this compound inhibited the inflammatory response induced by the spike protein in vitro on Huh7.5 and A549 epithelial cells [19].

Moreover, to confirm the attractive potential of nanomedicine, it should be noted that all the clinical studies described above used nanoformulations of curcumin to enhance its efficacy [21,22,23,24,25,26,27,28,29,30,31].

Another strategy involves the employment of curcumin as photosensitizer in a photodynamic therapy setting.

Indeed, when testing the photodynamic approach, a >99% reduction in the RNA viral load at 7 days post-infection/treatment was detected when the virus and the curcumin were irradiated and then transferred to the cell culture with concentrations of curcumin that were not antiviral by themselves (5 and 1 μM). Laser light could excite the photosensitizers (curcumin), then, through type I and II mechanisms, the energy may be transferred to O_2_, forming short-lived reactive oxygen species (ROS) at the sites of irradiation, mainly singlet oxygen, superoxide radical anions, hydrogen peroxide, and hydroxyl radicals. ROS in turn can damage the microorganism, resulting in oxidation of the lipids and protein forming the external surface of virions, but also targeting nucleic acids [41].

Prior to our work, a nanoformulation of curcumin was tested with a laser light at 450 nm against SARS-CoV-2-positive plasma [20]. The doses of irradiation (output irradiance of 1.6 W/cm^2^ and energy densities of 104.5, 313.7, and 522.8 J/cm^2^) were higher compared to those employed in the current study and the experimental settings were different, with plasma versus in vitro irradiation of the cell culture medium containing the virus. Pourhajibagher at al. [20] showed a complete recovery of cell survival and no apoptotic effect when Vero E6 cells were inoculated with PDT-processed plasma. Moreover, the treatment did not negatively influence the characteristics of plasma, such as total plasma protein content, anti-A/anti-B antibody titers, and coagulation test results. Instead, our data showed that a greater reduction of the infectious fraction of SARS-CoV-2 can also be achieved using lower doses of laser light; indeed, 0.25 W/cm^2^ and 30 J/cm^2^ (CW) were sufficient to cause viral inactivation in the presence of curcumin.

Our study presents some limitations, ascribable mainly to the in vitro experimental setting employed. Indeed, in vitro studies present a major drawback related to the application of the results in a real-life in vivo context. Our data highlighted that curcumin and curcumin-based PDT were effective only when the treatment was delivered prior to the cellular inoculation; therefore, this virucidal therapy can be exploited in the disinfection of biological fluids (e.g., plasma), but further studies are needed to confirm the real impact of curcumin on patients. In fact, the results of previous clinical trials indicated immunomodulatory effects, rather than virucidal action [21,22,23,24,25,26,27,28,29,30,31]. This restricted activity may be also due to the bioavailability of pure curcumin, and nanomedicine can help in overcoming this issue [20]. Indeed, all the studies conducted to date on COVID-19 patients in this area of research have employed curcumin nanoformulations [21,22,23,24,25,26,27,28,29,30,31].

Moreover, the exploitation of a virus isolated from clinical samples limited the analyses that were conducted in the present study due to the need to work in a BLS3 facility. TEM analysis and antigen-based tests could confirm the results obtained with the RNase protection assay. Thus, in future studies on viral photodynamic inactivation, other pseudotype models may be utilized employing pseudoviral particles, as recently reported by Sadraeian et al. [42].

## 5. Conclusions

Our results show that curcumin is an efficacious direct antiviral agent, especially when the treatment is performed prior to virus inoculation; moreover, we reduced the curcumin concentration, while at the same time maintaining antiviral activity. Raw curcumin, without manipulation in a nanoformulation, can be exploited as a photosensitizer coupled with blue laser light in a photodynamic strategy. PDT possesses great advantages compared to traditional drug-based therapies. As it is not target-specific, it presents higher flexibility and a broad spectrum of activity against different viruses (and virus variants). PDT can be exploited for different purposes, ranging from blood product (e.g., plasma) decontamination and surface and liquid disinfection to applications on human infections.

## Data Availability

All the data used to support the findings in this study are included in the article.

## References

[1] Hewlings S.J., Kalman D.S. (2017). Curcumin: A Review of Its Effects on Human Health. Foods.

[2] Gopi S., Jacob J., Varma K., Jude S., Amalraj A., Arundhathy C.A., George R., Sreeraj T., Divya C., Kunnumakkara A.B. (2017). Comparative Oral Absorption of Curcumin in a Natural Turmeric Matrix with Two Other Curcumin Formulations: An Open-label Parallel-arm Study: Comparative Oral Absorption of Curcumin. Phytother. Res..

[3] Santini A., Tenore G.C., Novellino E. (2017). Nutraceuticals: A paradigm of proactive medicine. Eur. J. Pharm. Sci..

[4] Kaenkumchorn T., Kesavan A. (2019). Dietary Management of Pediatric Inflammatory Bowel Disease. J. Med. Food.

[5] Zhai K., Brockmüller A., Kubatka P., Shakibaei M., Büsselberg D. (2020). Curcumin’s Beneficial Effects on Neuroblastoma: Mechanisms, Challenges, and Potential Solutions. Biomolecules.

[6] Moore K. (2018). N-Acetyl Cysteine and Curcumin in Pediatric Acute-Onset Neuropsychiatric Syndrome. J. Child Adolesc. Psychopharmacol..

[7] Elad S., Meidan I., Sellam G., Simaan S., Zeevi I., Waldman E., Weintraub M., Revel-Vilk S. (2013). Topical curcumin for the prevention of oral mucositis in pediatric patients: Case series. Altern. Ther. Health Med..

[8] Tenero L., Piazza M., Zanoni L., Bodini A., Peroni D., Piacentini G.L. (2016). Antioxidant supplementation and exhaled nitric oxide in children with asthma. Allergy Asthma Proc..

[9] Miserocchi E., Giuffrè C., Cicinelli M.V., Marchese A., Gattinara M., Modorati G., Bandello F. (2020). Oral phospholipidic curcumin in juvenile idiopathic arthritis-associated uveitis. Eur. J. Ophthalmol..

[10] Ellerkamp V., Bortel N., Schmid E., Kirchner B., Armeanu-Ebinger S., Fuchs J. (2016). Photodynamic Therapy Potentiates the Effects of Cur-cumin on Pediatric Epithelial Liver Tumor Cells. Anticancer Res..

[11] Sorg C., Schmid E., Bortel N., Fuchs J., Ellerkamp V. (2020). Antitumor effects of curcumin in pediatric rhabdomyosarcoma in combination with chemotherapy and phototherapy in vitro. Int. J. Oncol..

[12] Rupel K., Zupin L., Brich S., Mardirossian M., Ottaviani G., Gobbo M., Di Lenarda R., Pricl S., Crovella S., Zacchigna S. (2021). Antimicrobial activity of amphiphilic nanomicelles loaded with curcumin against *Pseudomonas aeruginosa* alone and activated by blue laser light. J. Biophotonics.

[13] Adamczak A., Ożarowski M., Karpiński T.M. (2020). Curcumin, a Natural Antimicrobial Agent with Strain-Specific Activity. Pharmaceuticals.

[14] Jennings M.R., Parks R.J. (2020). Curcumin as an Antiviral Agent. Viruses.

[15] Goc A., Sumera W., Rath M., Niedzwiecki A. (2021). Phenolic compounds disrupt spike-mediated receptor-binding and entry of SARS-CoV-2 pseudo-virions. PLoS ONE.

[16] Marín-Palma D., Tabares-Guevara J.H., Zapata-Cardona M.I., Flórez-Álvarez L., Yepes L.M., Rugeles M.T., Zapata-Builes W., Hernandez J.C., Taborda N.A. (2021). Curcumin Inhibits In Vitro SARS-CoV-2 Infection In Vero E6 Cells through Multiple Antiviral Mechanisms. Molecules.

[17] Bormann M., Alt M., Schipper L., van de Sand L., Le-Trilling V.T.K., Rink L., Heinen N., Madel R.J., Otte M., Wuensch K. (2021). Turmeric Root and Its Bioactive Ingredient Curcumin Effectively Neutralize SARS-CoV-2 In Vitro. Viruses.

[18] Goc A., Rath M., Niedzwiecki A. (2022). Composition of naturally occurring compounds decreases activity of Omicron and SARS-CoV-2 RdRp complex. Eur. J. Microbiol. Immunol..

[19] Sharma V.K., Prateeksha, Singh S.P., Singh B.N., Rao C.V., Barik S.K. (2022). Nanocurcumin Potently Inhibits SARS-CoV-2 Spike Protein-Induced Cytokine Storm by Deactivation of MAPK/NF-κB Signaling in Epithelial Cells. ACS Appl. Bio. Mater..

[20] Pourhajibagher M., Azimi M., Haddadi-Asl V., Ahmadi H., Gholamzad M., Ghorbanpour S., Bahador A. (2021). Robust antimicrobial photodynamic therapy with curcumin-poly (lactic-co-glycolic acid) nanoparticles against COVID-19: A preliminary in vitro study in Vero cell line as a model. Photodiagnosis Photodyn. Ther..

[21] Ahmadi R., Salari S., Sharifi M.D., Reihani H., Rostamiani M.B., Behmadi M., Taherzadeh Z., Eslami S., Rezayat S.M., Jaafari M.R. (2021). Oral nano-curcumin formulation efficacy in the management of mild to moderate outpatient COVID-19: A randomized triple-blind placebo-controlled clinical trial. Food Sci. Nutr..

[22] Asadirad A., Nashibi R., Khodadadi A., Ghadiri A.A., Sadeghi M., Aminian A., Dehnavi S. (2022). Antiinflammatory potential of nano-curcumin as an alternative therapeutic agent for the treatment of mild-to-moderate hospitalized COVID-19 patients in a placebo-controlled clinical trial. Phytotherapy Res..

[23] Hassaniazad M., Eftekhar E., Inchehsablagh B.R., Kamali H., Tousi A., Jaafari M.R., Rafat M., Fathalipour M., Nikoofal-Sahlabadi S., Gouklani H. (2021). A triple-blind, placebo-controlled, randomized clinical trial to evaluate the effect of curcumin-containing nanomicelles on cellular immune responses subtypes and clinical outcome in COVID-19 patients. Phytotherapy Res..

[24] Shafie E.H., Taheri F., Alijani N., Okhovvat A.R., Goudarzi R., Borumandnia N., Aghaghazvini L., Rezayat S.M., Jamalimoghadamsiahkali S., Hosseinzadeh-Attar M.J. (2022). Effect of nanocurcumin supplementation on the severity of symptoms and length of hospital stay in patients with COVID-19: A randomized double-blind placebo-controlled trial. Phytotherapy Res..

[25] Khan A., Iqtadar S., Mumtaz S.U., Heinrich M., Pascual-Figal D.A., Livingstone S., Abaidullah S. (2022). Oral Co-Supplementation of Curcumin, Quercetin, and Vitamin D3 as an Adjuvant Therapy for Mild to Moderate Symptoms of COVID-19—Results From a Pilot Open-Label, Randomized Controlled Trial. Front. Pharmacol..

[26] Kishimoto A., Imaizumi A., Wada H., Yamakage H., Satoh-Asahara N., Hashimoto T., Hasegawa K. (2021). Newly Developed Highly Bioavailable Curcumin Formulation, curcuRougeTM, Reduces Neutrophil/Lymphocyte Ratio in the Elderly: A Double-Blind, Placebo-Controlled Clinical Trial. J. Nutr. Sci. Vitaminol..

[27] Pawar K.S., Mastud R.N., Pawar S.K., Pawar S.S., Bhoite R.R., Bhoite R.R., Kulkarni M.V., Deshpande A.R. (2021). Oral Curcumin with Piperine as Adjuvant Therapy for the Treatment of COVID-19: A Randomized Clinical Trial. Front. Pharmacol..

[28] Saber-Moghaddam N., Salari S., Hejazi S., Amini M., Taherzadeh Z., Eslami S., Rezayat S.M., Jaafari M.R., Elyasi S. (2021). Oral nano-curcumin formulation efficacy in management of mild to moderate hospitalized coronavirus disease-19 patients: An open label nonrandomized clinical trial. Phytotherapy Res..

[29] Tahmasebi S., El-Esawi M.A., Mahmoud Z.H., Timoshin A., Valizadeh H., Roshangar L., Varshoch M., Vaez A., Aslani S., Navashenaq J.G. (2021). Immunomodulatory effects of nanocurcumin on Th17 cell responses in mild and severe COVID-19 patients. J. Cell. Physiol..

[30] Tahmasebi S., Saeed B.Q., Temirgalieva E., Yumashev A.V., El-Esawi M.A., Navashenaq J.G., Valizadeh H., Sadeghi A., Aslani S., Yousefi M. (2021). Nanocurcumin improves Treg cell responses in patients with mild and severe SARS-CoV2. Life Sci..

[31] Valizadeh H., Abdolmohammadi-Vahid S., Danshina S., Gencer M.Z., Ammari A., Sadeghi A., Roshangar L., Aslani S., Esmaeilzadeh A., Ghaebi M. (2020). Nano-curcumin therapy, a promising method in modulating inflammatory cytokines in COVID-19 patients. Int. Immunopharmacol..

[32] Crovella S., de Freitas L.C., Zupin L., Fontana F., Ruscio M., Pena E.P.N., Pinheiro I.O., Junior T.C. (2022). Surfactin Bacterial Antiviral Lipopeptide Blocks In Vitro Replication of SARS-CoV-2. Appl. Microbiol..

[33] Araf Y., Akter F., Tang Y., Fatemi R., Alam Parvez S., Zheng C., Hossain G. (2022). Omicron variant of SARS-CoV-2: Genomics, transmissibility, and responses to current COVID-19 vaccines. J. Med. Virol..

[34] CDC COVID Data Tracker. https://covid.cdc.gov/covid-data-tracker.

[35] Henderson L.A., Canna S.W., Friedman K.G., Gorelik M., Lapidus S.K., Bassiri H., Behrens E.M., Ferris A., Kernan K.F., Schulert G.S. (2021). American College of Rheumatology Clinical Guidance for Multisystem Inflammatory Syndrome in Children Associated With SARS–CoV-2 and Hyperinflammation in Pediatric COVID-19: Version 2. Arthritis Rheumatol..

[36] Chen T.-Y., Chen D.-Y., Wen H.-W., Ou J.-L., Chiou S.-S., Chen J.-M., Wong M.-L., Hsu W.-L. (2013). Inhibition of Enveloped Viruses Infectivity by Curcumin. PLoS ONE.

[37] Anggakusuma, Colpitts C.C., Schang L.M., Rachmawati H., Frentzen A., Pfaender S., Behrendt P., Brown R.J.P., Bankwitz D., Steinmann J. (2013). Turmeric curcumin inhibits entry of all hepatitis C virus genotypes into human liver cells. Gut.

[38] Lee W.-H., Loo J.C.Y., Bebawy M., Luk F., Mason R.S., Rohanizadeh R. (2013). Curcumin and its Derivatives: Their Application in Neuropharmacology and Neuroscience in the 21st Century. Curr. Neuropharmacol..

[39] Moustapha A., Pérétout P.A., Rainey N.E., Sureau F., Geze M., Petit J.-M., Dewailly E., Slomianny C., Petit P.X. (2015). Curcumin induces crosstalk between autophagy and apoptosis mediated by calcium release from the endoplasmic reticulum, lysosomal destabilization and mitochondrial events. Cell Death Discov..

[40] Trigo-Gutierrez J.K., Vega-Chacón Y., Soares A.B., de Oliveira Mima E.G. (2021). Antimicrobial Activity of Curcumin in Nanoformulations: A Comprehensive Review. Int. J. Mol. Sci..

[41] Wiehe A., O’Brien J.M., Senge M.O. (2019). Trends and targets in antiviral phototherapy. Photochem. Photobiol. Sci..

[42] Sadraeian M., Junior F.F.P., Miranda M., Galinskas J., Fernandes R.S., da Cruz E.F., Fu L., Zhang L., Diaz R.S., Cabral-Miranda G. (2022). Study of Viral Photoinactivation by UV-C Light and Photosensitizer Using a Pseudotyped Model. Pharmaceutics.

